# Effectiveness of the ACA (Availability, Current issues and Anticipation) training programme on GP-patient communication in palliative care; a controlled trial

**DOI:** 10.1186/1471-2296-14-93

**Published:** 2013-07-02

**Authors:** Willemjan Slort, Annette H Blankenstein, Bart PM Schweitzer, Dirk L Knol, Luc Deliens, Neil K Aaronson, Henriëtte E van der Horst

**Affiliations:** 1 Department of General Practice & Elderly Care Medicine, EMGO + Institute for Health and Care Research, VU University Medical Center, Amsterdam, The Netherlands; 2 Department of Epidemiology and Biostatistics, EMGO + Institute for Health and Care Research, VU University Medical Center, Amsterdam, The Netherlands; 3 Department of Public and Occupational Health, EMGO + Institute for Health and Care Research, VU University Medical Center, Amsterdam, The Netherlands; 4 End-of-Life Care Research Group, Ghent University & Vrije Universiteit Brussel, Brussels, Belgium; 5 Division of Psychosocial Research and Epidemiology, The Netherlands Cancer Institute, Amsterdam, The Netherlands

**Keywords:** Communication, Controlled Clinical Trial, Education Medical Continuing, Family Practice, Palliative Care

## Abstract

**Background:**

Communicating effectively with palliative care patients has been acknowledged to be somewhat difficult, but little is known about the effect that training general practitioners (GPs) in specific elements of communication in palliative care might have. We hypothesized that GPs exposed to a new training programme in GP-patient communication in palliative care focusing on *availability* of the GP for the patient, *current issues* the GP should discuss with the patient and *anticipation* by the GP of various scenarios (ACA), would discuss more issues and become more skilled in their communication with palliative care patients.

**Methods:**

In this controlled trial among GPs who attended a two-year Palliative Care Peer Group Training Course in the Netherlands only intervention GPs received the ACA training programme. To evaluate the effect of the programme a content analysis (Roter Interaction Analysis System) was performed of one videotaped 15-minute consultation of each GP with a simulated palliative care patient conducted at baseline, and one at 12 months follow-up. Both how the GP communicated with the patient (‘availability’) and the number of current and anticipated issues the GP discussed with the patient were measured quantitatively. We used linear mixed models and logistic regression models to evaluate between-group differences over time.

**Results:**

Sixty-two GPs were assigned to the intervention and 64 to the control group. We found no effect of the ACA training programme on how the GPs communicated with the patient or on the number of issues discussed by GPs with the patient. The total number of issues discussed by the GPs was eight out of 13 before and after the training in both groups.

**Conclusion:**

The ACA training programme did not influence how the GPs communicated with the simulated palliative care patient or the number of issues discussed by the GPs in this trial. Further research should evaluate whether this training programme is effective for GPs who do not have a special interest in palliative care and whether studies using outcomes at patient level can provide more insight into the effectiveness of the ACA training programme.

**Trial registration:**

Current Controlled Trials ISRCTN56722368

## Background

While effective communication between health care professionals and patients is considered to be an essential requirement in order to provide high-quality care, [1-6] communicating with palliative care patients has been acknowledged as being more difficult than communicating with patients with less serious conditions [7]. Communication in palliative care involves addressing a complex mix of physical, psychosocial and spiritual/existential issues within the context of impending death. If a health care professional does not communicate as well as he could, some, if not many, of the problems that patients are facing might not be identified. Consequently, it is likely that the health care professional will not be able to take the appropriate actions, and the patient’s quality of life may be unnecessarily impaired.

Several studies have demonstrated the effectiveness of basic communication skills training programmes in improving oncologists’ or oncology nurses’ communication with oncology patients, including those receiving palliative care [8,9].

General practitioners (GPs) are trained in doctor-patient communication as part of their pre- and postgraduate education. However, this does not always cover specific training in communication with palliative care patients [10]. Little is known about the effectiveness of training GPs in specific elements of communication in palliative care.

To fill this gap, we designed a new training programme for GP-patient communication in palliative care, based on recent studies [8,11-13]. This programme, focusing on *availability* of the GP for the patient, *current issues* the GP should discuss with the patient, and *anticipation* by the GP of various scenarios (ACA), appeared to be applicable to GPs and GP trainees (see Tables 1 and 2) [14]. In this paper we report on a controlled clinical trial which evaluated the effectiveness of this ACA training programme on GP-patient communication in palliative care. We hypothesized that GPs exposed to the training programme would discuss more current and anticipated issues and would become more skilled in their communication with palliative care patients.

**Table 1:** **The eight steps of the ACA** (**availability**, **current issues**, **anticipation**) **training programme**

Step 1	*Videotaped GP-patient interview* with a trained actor simulating a patient in an advanced stage of lung (role A) or colon (role B) cancer, according to detailed scripts; immediately after the interview the participant receives general feedback on communication style from the actor.
Step 2	*Instructions on the ACA checklist,* using oral presentations and written information (ACA booklet).
Step 3	*Feedback according to the ACA checklist* on GP performance during the videotaped GP-patient interview in step 1.
Step 4	*Studying* the ACA checklist, *discussing* this material with peers in small groups, and *trying out* newly acquired skills in their own general practice to identify problem areas from their own experience.
Step 5	*Formulating learning goals* based on the previous steps.
Step 6	*Role-play exercises* tailored to the GP’s individual learning goals.
Step 7	A *second videotaped interview* with an actor simulating a patient.
Step 8	Using the second videotaped interview and the ACA checklist as tools for *self-assessment* of their communication skills.

**Table 2:** **The ACA** (**availability**, **current issues**, **anticipation**) **checklist**

***Availability****(of the GP for the patient):*	
1.	Taking time
2.	Allowing any subject to be discussed
3.	Active listening
4.	Facilitating behaviour (e.g. empathic, respectful, attentive, occasionally also phoning or visiting the patient spontaneously)
5.	Shared decision-making with regard to diagnosis and treatment plan
6.	Accessibility (e.g. phone numbers)
***Current issues****(that should be raised by the GP):*	
1.	Diagnosis
2.	Prognosis
3.	Patient’s complaints and worries: - physical
4.	- Psychosocial
5.	- Spiritual/existential
6.	Wishes for the present and the coming days
7.	Unfinished business, bringing life to a close
8.	Discussing treatment and care options (concerning 1-7)
***Anticipating****(various scenarios):*	
1.	Offering follow-up appointments
2.	Possible complications
3.	Wishes for the coming weeks/months (personal wishes as well as preferences with regard to medical decisions)
4.	The actual process of dying (final hours/days)
5.	End-of-life decisions

## Methods

### Setting and participants

This controlled trial was conducted in the context of an existing post-graduate two-year Palliative Care Peer Group Training Course (PCPT), consisting of four two-day residential courses, followed by two-hour peer group sessions with five GPs in each group, facilitated by a palliative care consultant, every six to eight weeks. All GPs enrolled in the four PCPT courses in 2006 and 2007 were invited to take part in the study. As our intervention was added to an existing training course, we had to assign whole training groups to either the intervention or the control condition. Because we wanted to start with an intervention group in 2006, and to prevent contamination between groups, GPs enrolled in the PCPT courses conducted in Eindhoven (2006) and Rotterdam (2007) were assigned to the intervention condition in which the ACA training programme was integrated into the PCPT course. GPs who enrolled in the PCPT courses in Amsterdam (both 2007) were assigned to the control condition in which the ACA training programme component was not included.

### Intervention

The development of the ACA training programme has been reported elsewhere [14]. The programme consists of eight steps (see Table 1) and is supported by the ACA checklist (see Table 2). Steps 1 and 2 took place on the first day of the training programme. Within two months all participants received individual feedback on their videotaped simulated consultation (step 3). During the following months they had to complete step 4 in order to formulate their personal learning goals (step 5). Six months after the start of the programme, the GPs participated in role-play exercises that were tailored to their learning goals (step 6). Finally, a second consultation with an actor simulating a patient was videotaped (step 7) to allow participants to assess their communication skills against the ACA checklist (step 8).

### Sample size

For calculating sample size, we used the outcome measure ‘number of issues discussed by the GP’ and considered a difference of 0.5 standard deviation (which corresponded with one extra issue discussed by the GP) between intervention and control conditions as a clinically relevant difference. Such a difference can be detected with 64 GPs in each group (power 0.80, two-sided alpha 0.05).

### Outcome measures

Outcome measures of this study were determined in discussion with a panel of experts in palliative care research. We decided to measure both how the GP communicated with the patient and what he discussed with him. These outcomes fit in well with the content of the ACA training programme on how to communicate with the patient (availability items) and what to discuss (the current and anticipated issues). Both ‘how’ and ‘what’ were measured quantitatively.

The number of issues discussed (‘what’) was defined as the summed number of 13 current and anticipated issues about which the GP made at least one utterance concerning that issue, during the simulated consultation. Additionally, we calculated for each issue the percentage of consultations in which the GPs discussed that issue.

The quality of a GP’s communicative behaviour ('how’) was defined as their scores on the six availability items. Because this complex outcome consisted of several numbers and percentages its sub-scores could not be summed up and were reported separately. Additionally, verbal dominance was calculated to evaluate whether the training influenced the GP’s dominance during the consultation.

### Measurement instrument

To measure both outcomes (‘how’ and ‘what’) from the videotaped consultations we used the Roter Interaction Analysis System (RIAS) [15,16]. The RIAS, which was developed in the United States, has been used successfully in previous studies in Dutch general practice settings [17]. It distinguishes mutually exclusive and exhaustive categories into which verbal utterances that convey a complete thought can be classified. A distinction is made between instrumental or task-oriented categories, and affective or socio-emotional categories. Task-oriented categories refer to utterances that address a patient’s physical or psychosocial problems. Affective categories carry explicit emotional content and refer to aspects of communication that are needed to establish a therapeutically effective relationship. The RIAS also rates ‘global affects’ on 6-point scales (e.g. friendliness/warmth)

For the outcome ‘number of issues discussed’ we added the current and anticipated issues to the task-oriented categories of the original RIAS. For the outcome quality of GP’s communicative behaviour we added several study-specific 6-point scales to the RIAS (e.g. the extent to which the GP took time with the simulated patient). Four of the six availability items could be scored positively (e.g. ‘taking time’) as well as negatively (e.g. ‘not taking time’). As we were especially interested in the communication by the GPs, we only calculated scores for the GPs (and not for the simulated patient).

### Measurement procedure

For each GP participating in the study, we videotaped a 15-minute consultation with a simulated palliative care patient at baseline and at follow-up. The baseline assessment took place on the first day of the course; the follow-up assessment 12 months later, halfway through the two-year PCPT course. At baseline, half of the GPs from each of the four PCPT courses had a consultation with a trained actor who role-played a patient with advanced stage lung cancer. The other 50% had a consultation with an actor playing the role of a patient with advanced colon cancer. At the follow-up assessment, the simulated patient to whom the GPs were assigned was reversed from the baseline assessment. The setting in which the consultation took place was standardized to avoid any environmental variability.

The participating GPs were aware of their group allocation, but the actors involved in role-playing a palliative care patient and those who rated the videotaped GP-simulated patient encounters were not.

### Coding procedure

Coding was carried out directly from videotape by four trained raters using The Observer® software (http://www.noldus.com/). Average coding time was three to four times the duration of the consultation. Throughout the coding period, a random sample of 11.5% of the tapes was rated by all coders to assess interrater reliability. Interrater reliability averaged for the ACA issues 0.85 (range 0.68-0.99) and for the percentages of utterances with a mean occurrence greater than 2% 0.71 (range 0.56-0.89), respectively [15,16]. These reliability estimates are comparable to those achieved in other studies [18-21].

### Statistical analysis

We assessed the comparability of the GPs in the intervention and the control condition with regard to socio-demographic and professional characteristics using the Chi-square statistic for categorical variables and the Mann–Whitney test for continuous variables. Variables on which the two groups were not comparable at baseline were entered as covariates in subsequent multivariable analyses.

We summed the number of 13 current and anticipated issues that were discussed by the GP during the simulated consultation. Consequently, the scale ranged from 0 to 13. For each issue we calculated the percentage of consultations in which that issue was discussed. For the outcome quality of communicative behaviour we calculated mean numbers and percentages of the several sub-scores. Verbal dominance was calculated by dividing the sum of all GP utterances by the sum of all patient utterances.

We used linear mixed models and accompanying effect sizes to evaluate between-group differences over time for interval level outcome variables (e.g. mean numbers and percentages). For dichotomous outcome variables (e.g. whether a given issue was discussed) we used the logistic regression method of generalized estimating equations (GEE) to account for dependence of data due to repeated measures, yielding odds ratios. In all analyses we used the GP’s sex, years of experience as GP, urban versus rural or semi-rural practice location, the actor, and duration of the consultation longer than 15 minutes as covariates. In order to adjust for multiple testing, the level of significance was set at 0.01. All data were entered and analysed in SPSS 20.0 (SPSS, Inc., Chicago, IL).

## Results

### GP characteristics

**Figure 1:**
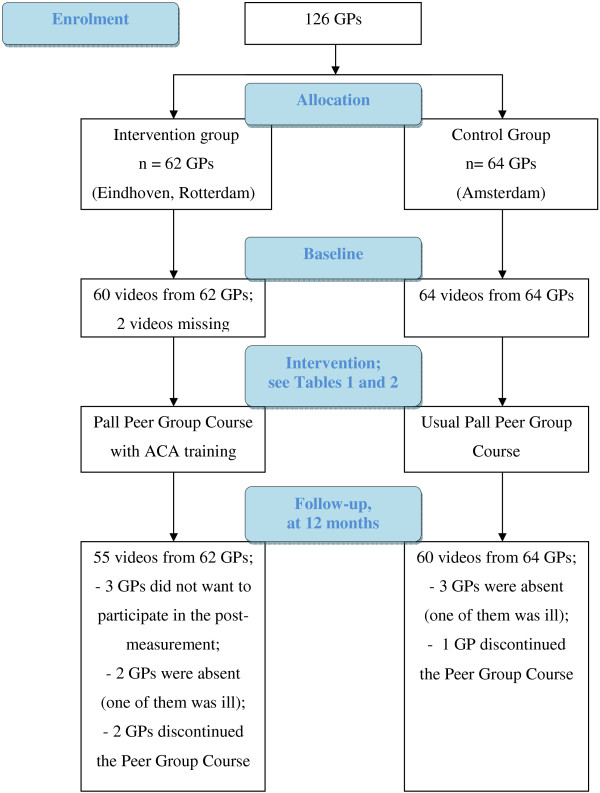
ACA trial Consort flow diagram.

All 126 GPs eligible for this study agreed to participate. Sixty-two were assigned to the intervention and 64 to the control group (see Figure 1). GPs in the intervention group were less likely to practise in an urban location and had a few more years of experience than those in the control group. No further significant between-group differences were observed (Table 3).

**Table 3:** **Socio**-**demographic and professional characteristics of participating general practitioners** (**GPs**)

**Characteristics of participating GPs**	**Intervention group;** **n=62 GPs**	**Control group;** **n=64 GPs**	**P**
Gender female, n (%)	28 (45%)	38 (59%)	.15
Age (years)*	49 (33–60)	48 (33–61)	.23
Years of experience as a GP*	16 (1–34)	14 (1–32)	.**034**
Group or single-handed practice			.98
- Group practice, n (%)	24 (39%)	24 (39%)	
- Duo practice, n (%)	23 (37%)	24 (36%)	
- Single-handed practice, n (%)	15 (24%)	16 (25%)	
Practice location urban (versus rural/semi-rural), n (%)	22 (35%)	44 (69%)	< .**001**
Working percentage of FTE*	.80 (.50-1.00)	.75 (.40-1.00)	.06
Vocational GP trainers, n (%)	17 (27%)	19 (30%)	.84
Courses in palliative care attended by GP during the previous two years, n (%)	31 (50%)	37 (58%)	.47

Data are presented as number (percentage) or * median (range); P= p-value using chi square test or Mann Whitney tests as appropriate.

### Number of issues discussed (‘what’)

We found no statistically significant differences over time between the intervention and control group in the mean total number of ACA issues, the mean number of current issues or the mean number of anticipated issues discussed (Table 4). In the total study sample, GPs raised on average eight of the 13 ACA issues during the consultation with the simulated palliative care patient (4.5 current and 3.5 anticipated issues).

**Table 4:** **Number and type of current and anticipated issues that the GPs addressed during the simulated 15**-**minute consultations in intervention group** (**n**=**62**) **and control group** (**n**=**64**)

**ACA issues**	**Baseline**		**Follow-up**		**Effect(difference**^**3**^**or odds ratio**^**4**^**) (95%confidence interval)**	**P-value**
	**Intervention n=60**	**Control n=64**	**Intervention n=55**	**Control n=60**		
**All current and anticipated issues(0–13)**^**1**^	8.00 (1.46)	7.80 (1.84)	8.05 (1.69)	7.78 (1.63)	-.07 (−.82; .69) ^3^	.86
**Current issues(0–8)**^**1**^	4.48 (.87)	4.58 (1.05)	4.73 (1.10)	4.52 (1.11)	.29 (−.20; .78) ^3^	.24
1. Diagnosis ^2^	43	50	42	57	.76 (.25; 2.26) ^4^	.63
2. Prognosis ^2^	60	55	65	53	1.23 (.45; 3.36) ^4^	.69
3. Complaints, physical ^2^	100	100	100	100	- ^5^	-
4. Complaints, psychosocial ^2^	100	98	100	98	- ^5^	-
5. Complaints, spiritual/existential ^2^	7	2	2	7	.06 (.001; 3.03) ^4^	.16
6. Wishes, at present ^2^	33	39	35	32	1.53 (.52; 4.53) ^4^	.44
7. Unfinished business ^2^	10	17	29	13	5.81 (1.32; 25.61) ^4^	.020
8. treatment and care options ^2^	95	97	100	92	- ^5^	-
**Anticipated issues (0–5)**^**1**^	3.52 (1.20)	3.22 (1.24)	3.33 (1.17)	3.27 (1.31)	-.39 (−.98; .20) ^3^	.19
1. Follow-up appointments ^2^	93	92	100	90	- ^5^	-
2. Possible complications ^2^	73	72	60	70	.47 (.14; 1.63) ^4^	.23
3. Wishes, for the coming months ^2^	85	78	93	77	2.12 (.41; 10.94) ^4^	.37
4. The actual process of dying ^2^	50	48	53	50	.82 (.27; 2.45) ^4^	.72
5. End-of-life decisions ^2^	50	31	27	40	.**13** (.**03**; .**50**) ^4^	.**003**

^1^ Observed mean (and standard deviation) of the number of issues the GP addressed during the consultation by at least one utterance concerning an issue; interrater reliability for the ACA issues averaged 0.85 (range 0.68-0.99).

^2^ Observed percentage of consultations in which a GP made at least one utterance concerning this issue.

^3^ Effect of intervention (95% confidence interval) using a linear mixed model, adjusted for baseline differences (years of experience as GP and urban versus rural/semi-rural practice location) and for possible influences of the GP’s sex, several actors simulating palliative care patients, and duration of the consultations longer than 15 minutes.

^4^ Odds ratio (95% confidence interval) using a logistic regression (generalized estimating equations=GEE) model, adjusted for baseline differences (years of experience as GP and urban versus rural/semi-rural practice location) and for possible influences of the GP’s sex, several actors simulating palliative care patients, and duration of the consultations longer than 15 minutes.

^5^ The logistic regression (GEE) model is not fit for results of binomial data close to 0 or 100% respectively.

### Different issues discussed

A significant between-group difference over time was found only in the percentage of consultations in which the anticipated issue ‘end-of-life decisions’ was discussed: the percentage of consultations in which this issue was discussed decreased from 50% at baseline to 27% at follow-up in the intervention group, while an increase from 31% to 41% was seen in the control group (Table 4).

The four ACA issues physical complaints, psychosocial complaints, discussing treatment and care options and offering follow-up appointments were addressed in 90-100% of the consultations in both groups at baseline and follow-up measurements. Spiritual/existential issues and ‘unfinished business’ were infrequently addressed by the GPs.

### Quality of communicative behaviour (‘how’)

No statistically significant between-group differences over time were observed in any of the outcomes related to availability, with the exception of the task-focused utterance ‘check’ (Table 5).

**Table 5:** **Scores on the availability items** (‘**communicative behaviour**’) **during the simulated 15**-**minute consultations of GPs in intervention group** (**n**=**62**) **and control group** (**n**=**64**)

**Availability items**	**Baseline**		**Follow-up**		**Effect(difference**^**7**^**or odds ratio**^**8**^**) (95%confidence interval)**	**P-value**
**POSITIVE**	**Intervention n=60**	**Control n=64**	**Intervention n=55**	**Control n=60**		
**1**. ***Taking time*** (3 GARs, 3–18)^1^	13.23 (2.35)	13.05 (2.15)	12.95 (2.63)	12.40 (2.42)	.21 (−1.03; 1.46) ^7^	.73
**2**. ***Allowing any subject to be discussed*** (2 GARs, 2–12)^1^	8.45 (1.60)	8.55 (1.67)	8.38 (1.63)	8.12 (1.69)	.31 (−.55; 1.17) ^7^	.47
**3**. ***Active listening***						
A. Open/Closed Questions Ratio^2^	.65 (1.00)	.73 (1.12)	.57 (.44)	.58 (.74)	.07 (−.37; .52) ^7^	.74
B. Affective utterances (RIAS):						
1. Back-channel responses^3^	29.5 (11.4)	30.8 (11.5)	31.9 (8.7)	32.8 (12.6)	.31 (−3.10; 3.71) ^7^	.86
2. Shows approval (=approval +compliment)^4^	.49 (.79)	.33 (.53)	.52 (.91)	.58 (.89)	-.22 (−.64; .19) ^7^	.29
3. Verbal attention (= empathy + legitimizes + partnership)^4^	4.33 (2.87)	4.96 (3.53)	4.46 (3.35)	4.36 (2.79)	.81 (−.66; 2.27) ^7^	.28
4. Shows concern or worry^4^	.04 (.23)	.11 (.53)	.00 (.00)	.08 (.40)	-.004 (−.20; .19) ^7^	.96
5. Reassurance (e.g. reassures, encourages, shows optimism)^4^	1.24 (2.26)	.84 (1.32)	1.17 (1.69)	1.23 (1.53)	-.66 (−1.49; .17) ^7^	.12
6. Agreement (shows agreement or understanding)^4^	1.51 (1.61)	1.45 (2.09)	1.56 (1.49)	2.03 (2.44)	-.56 (−1.30; .17) ^7^	.13
7. Personal remarks, laughs^4^	4.25 (2.60)	5.50 (2.86)	4.03 (1.91)	5.17 (2.25)	.19 (−.97; 1.35) ^7^	.75
8. Silence^5^	12	17	34	33	1.55 (.43; 5.62) ^8^	.51
C. Task-focused utterances (RIAS):^4^						
1. Check (paraphrase/checks for understanding)	4.68 (2.91)	6.84 (4.33)	5.53 (3.74)	5.24 (3.36)	**2**.**60** (.**92**; **4**.**29**) ^7^	.**003**
2. Gives orientation, instructions, introduction	2.72 (3.02)	3.25 (3.07)	3.13 (2.26)	3.08 (2.82)	.60 (−.80; 2.01) ^7^	.40
3. Bids for repetition	.30 (0.84)	.27 (1.13)	.16 (.37)	.18 (.51)	-.05 (−.44; .35) ^7^	.82
4. Asks for understanding	.06 (.23)	.06 (.23)	.04 (.22)	.01 (.10)	.02 (−.09; .12) ^7^	.72
5. Asks for opinion	1.43 (1.14)	1.49 (1.23)	1.37 (1.21)	1.31 (1.14)	.17 (−.40; .75) ^7^	.55
**4**. ***Facilitating behaviour***						
A. Facilitating behaviour (5 GARs, 5–30)^1^	22.15 (3.28)	21.92 (3.70)	22.29 (3.50)	21.17 (3.62)	.65 (−.99; 2.30) ^7^	.43
B. Meta-communication^5^	22	16	22	15	.98 (.29; 3.33) ^8^	.97
**5**. ***Shared decision making with regard to diagnosis and treatment plan***						
A. Shared Decision Making (3 GARs, 3–18)^1^	11.77 (2.22)	12.13 (2.58)	11.80 (2.36)	11.22 (2.31)	.88 (−.37; 2.14) ^7^	.17
B. Extent of shared decision making (Range per topic 1–4)^6^	2.14 (.54)	2.22 (.57)	2.23 (.56)	2.16 (.57)	.14 (−.16; .45) ^7^	.35
**6**. ***Accessibility***^5^	10	12	11	12	1.03 (.20; 5.34) ^8^	.97
**NEGATIVE**						
**1**. ***Not taking time*** Hurried/Rushed (1 GAR, 1–6)^1^	2.60 (1.37)	2.80 (1.16)	2.53 (1.34)	2.62 (1.33)	.14 (−.52; .79) ^7^	.68
**2**. ***Not allowing a subject to be discussed*** disregard^5^	15	3	7	5	.24 (.02; 3.24) ^8^	.28
**3**. ***Not listening actively*** disagreement (=shows disapproval, criticism)^5^	0	0	2	2	- ^9^	−^9^
**4*Not facilitating behaviour*** (2 GARs, 2–12)^1^	2.37 (.74)	2.30 (.61)	2.24 (.58)	2.35 (.71)	-.19 (−.51; .14) ^7^	.26

^1^ Observed mean rating (and standard deviation) of a (or of the sum of some) Global Affect Rating(s) (GARs) for the GP; the scale of each Global Affect Rating ranges from 1 to 6; interrater reliability of the GARs averaged 0.19 (range 0–0.39; these ICCs were rather low due to low variances in the GARs between consultations); 3 GARs ‘taking time’: calmness, speaking quietly, and showing involvement; 2 GARs ‘allowing any subject to be discussed’: GP’s open attitude and allowing any subject to be discussed; 5 GARs ‘facilitating behaviour’: interest/attentiveness, friendliness/warmth, responsiveness/engagement, sympathetic/empathetic, and respectfulness; 3 GARs ‘shared decision making’: with regard to treatment and care options taking patient’s quality of life and meaningfulness into consideration, informing patient adequately, and involving patient in decisions about treatment and care options; 1 GAR ‘not taking time’: (hurried/rushed); and 2 GARs ‘not facilitating behaviour’: anger/irritation and anxiety/nervousness.

^2^ Observed mean ratio (and standard deviation) of the total number of GP’s open questions divided by the total number of GP’s closed questions during a consultation; because at baseline in the intervention group two GPs scored respectively 27 and 33 while the range of the other scores was from 0 to 5.67, we replaced these two outlying scores by the third to highest score (namely 5.67) to prevent a disproportional influence of these two scores on the mean ratio.

^3^ Observed mean percentage (and standard deviation) of the total number of back channels by the GP divided by the total number of all utterances (including the back-channels) by the GP during a consultation; interrater reliability of the scores on the RIAS utterance back channel was 0.89.

^4^ Observed mean percentage (and standard deviation) of the total number of this type of utterance by the GP divided by the total number of all utterances (with the exception of the back-channels) by the GP during a consultation (the back-channels were excepted to prevent dominance of all results by the rather high en variable number of back-channels that were scored during the consultations when compared to the numbers of all other utterances); interrater reliability of the scores on the four RIAS utterances with a mean occurrence greater than 2% (verbal attention, personal remarks, check, and giving orientation) averaged 0.66 (range 0.56-0.75).

^5^ Observed percentage of consultations of the intervention and control group at baseline and post-measurement in which the GP made at least one utterance concerning this issue.

^6^ Observed mean ratio (and standard deviation) of the sum of the ratings for the extent to which the GP had discussed the treatment or care options concerning the addressed problems with the patient (= shared decision making, rating 1 to 4) divided by the number of problems that were addressed during the consultation.

^7^ Effect of intervention (95% confidence interval) using a linear mixed model, adjusted for baseline differences (years of experience as GP and urban versus rural/semi-rural practice location) and for possible influences of the GP’s sex, several actors simulating palliative care patients, and duration of the consultations longer than 15 minutes.

^8^ Odds ratio (95% confidence interval) using a logistic regression (GEE) model, adjusted for baseline differences (years of experience as GP and urban versus rural/semi-rural practice location) and for possible influences of the GP’s sex, several actors simulating palliative care patients, and duration of the consultations longer than 15 minutes.

^9^ The logistic regression (GEE) model is not fit for results of binomial data close to 0 or 100% respectively.

Verbal dominance showed no significant between-group difference over time (P=0.6), with or without inclusion of the rather frequently scored back channels (=utterances indicating attentive listening, such as ‘mmm-huh’). In both groups the verbal dominance was about 1 and decreased slightly from baseline to follow-up (i.e. GPs became slightly less dominant in terms of proportion of given utterances).

## Discussion

In this controlled trial we found no significant effect of the ACA training programme on the total number of current and anticipated issues that GPs discussed in consultations with simulated palliative care patients, or on the quality of their communicative behaviour.

The total number of issues discussed by the GPs was eight out of 13 before and after the training in both groups. We consider this a rather high number during a 15-minute consultation. It may be that the high scores at baseline allowed little room for improvement on this outcome. This possible ceiling effect could be related to the fact that all GPs in this study were participating in a two-year Palliative Care Peer Group Training Course (PCPT), and probably had a more than average commitment to palliative care.

The results indicate that the frequency with which GPs exposed to the training programme discussed ‘end-of-life decisions’ actually declined over time, while it increased in the control group. For this finding and for the significant difference in the task-focused utterance ‘check’ we have no explanation other than that these are coincidental. The current issue ‘patient’s spiritual/existential complaints and worries’ was seldom discussed by the GPs, and did not change over time. This reflects findings from previous studies that GPs do not always consider discussing spiritual issues as part of their professional competence or responsibility [22].

Although we developed an evidence based intervention and used sound methods to evaluate its effectiveness, we found no effect on how and what the GP discussed with the simulated palliative care patient. Besides a possible ceiling effect in this group of GPs with more than average interest in palliative care, we considered also other possible explanations for these ‘negative’ results. The intervention might not have been effective or the outcome measures might not have been sensitive to change over time. Although the ACA checklist provides a concise summary of the essential factors for GP-patient communication in palliative care, all separate items (‘how’) and issues (‘what’) are not new, especially not for experienced GPs. Our quantitative content analysis (RIAS) of the consultations might not be sensitive enough in assessing overall quality of the GP’s communication with the patient. Although we discussed extensively the best outcomes for this intervention, in retrospect we doubt whether the number of issues discussed by the GP is an appropriate indicator of quality of communication. It might be that the GP discussed the same number of issues at baseline and at follow-up, but discussed these issues in a better way at follow-up. However, we also failed to detect a significant effect on the ‘how’ of GP-patient communication. Although we included the several actors who role-played a patient with advanced stage cancer in our analyses as a covariate, this factor might have influenced our results more than we could identify.

### Strengths and limitations of this study

To our knowledge, this is the first study on effectiveness of a communication training programme specifically targeted at GP-patient communication in palliative care [12]. Our intervention largely meets the recommendations for communication skills training in oncology as formulated at a recent consensus meeting by Stiefel et al. [23]. Both educational approach and content of the intervention are evidence-based [14]. The outcomes of our trial were based on behavioural observations of simulated GP-patient consultations assessed by a validated quantitative instrument (RIAS).

As we had to assign participating GPs to either the intervention or the control condition without randomization, we carefully compared both groups and included significant between-group differences on background characteristics as covariates in the subsequent analyses. The GPs were not blind to their training condition. As a trial with videotaped consultations of GPs with real palliative care patients was not deemed to be feasible, we used trained actors to simulate patients with advanced stage cancer. Our study was based on the four levels of competence according to the pyramid model of Miller; 1. knows (knowledge), i.e. recall of basic facts, principles, and theories; 2. knows how (applied knowledge), i.e. ability to solve problems, make decisions, and describe procedures; 3. shows how (performance), i.e. demonstration of skills in a controlled setting; and 4. does (action), i.e. behaviour in real practice [24]. We focused our effectiveness evaluation on the third level. Moreover, we measured one 15-minute consultation, while in daily practice, Dutch GPs visit their palliative care patients frequently at home and thus discussion of the 13 issues will be spread over several visits.

### Comparison with existing literature

We found no effectiveness studies that specifically address GP-patient communication in palliative care [12]. Two systematic reviews on effectiveness of communication training programmes for health professionals in cancer care reported positive effects (e.g. more open questions, expressions of empathy) from such training programmes [8,9]. These health professionals (not GPs) had probably received less extensive training in doctor-patient communication as part of their educational curriculum, and therefore the baseline level of their communication skills might have allowed more room for improvement compared with the GPs in our trial. Furthermore, these studies focused primarily on ‘breaking bad news’ and ‘dealing with patients’ feelings’ surrounding diagnosis, prognosis, and treatment options, while the ACA programme is targeted at issues in palliative care and anticipating the patient’s end-of-life concerns. In previous studies the primary outcomes were typically basic communication skills such as the availability aspects of the ACA checklist, while our primary outcome included the number of current and anticipated issues discussed by GPs. In their monograph on patient-centred communication in cancer care, Epstein and Street emphasize communication skills (i.e., how to provide information) more than specific issues to be addressed [25]. In their systematic review, Parker et al. discuss in detail the specific content as well as the style of end-of-life communication; the content areas they cover are similar to those of the ACA checklist [26]. However, the ACA checklist lays more emphasis on the patient’s personal wishes, unfinished business and bringing life to a close.

## Conclusion

In this trial with a specific group of GPs, the ACA training programme did not influence how the GPs communicated with the simulated palliative care patient or the number of issues discussed by the GPs. Further research should evaluate whether this training programme is effective for GPs who do not have a special interest in palliative care. Moreover, a study using outcomes at patient level might provide more insight into the effectiveness of the ACA training programme.

## Competing interests

The funding bodies had no involvement in or influence on the study, and the authors report no conflicts of interests to be declared.

## Authors’ contributions

WS, AHB, HEvdH, LD and NKA developed the intervention and the design of the study. WS collected the data. WS and DLK analyzed the data. WS and AHB drafted the manuscript. All authors made substantial contributions to the interpretation of the data and were involved in drafting the manuscript. All authors read and approved the final manuscript.

## Pre-publication history

The pre-publication history for this paper can be accessed here:

http://www.biomedcentral.com/1471-2296/14/93/prepub
